# The Nutrition Knowledge Level and Influencing Factors among Chinese Women Aged 18–49 Years in 2021: Data from a Nationally Representative Survey

**DOI:** 10.3390/nu15092034

**Published:** 2023-04-23

**Authors:** Yujie Qiu, Caicui Ding, Yan Zhang, Fan Yuan, Weiyan Gong, Ying Zhou, Chao Song, Jingwen Feng, Wenbin Zhang, Ailing Liu

**Affiliations:** National Institute for Nutrition and Health, Chinese Center for Disease Control and Prevention, Beijing 100050, China

**Keywords:** nutrition knowledge, awareness rate, influencing factors, women of childbearing age, multilevel logistic regression model

## Abstract

Women aged 18–49 years are women of childbearing age. Their nutrition knowledge (NK) is not only related to their physical health but also affects the diet quality of the entire family and the health status of the next generation. Studies that assess the NK level of women of childbearing age using representative data in China are lacking. This study aimed to evaluate the NK level of Chinese women aged 18–49 years and explore influencing factors at both the individual and provincial levels. Data were from the Chinese Nutrition and Health Knowledge Survey 2021. A total of 38,065 females aged 18–49 years were included in the analysis. A face-to-face validated standard questionnaire was used to collect NK from respondents. The full score of the questionnaire was 100. Respondents who scored higher than 75 were considered to have NK. The Rao-Scott chi-square test was used to compare the differences in the NK awareness rate between or among the subgroups. A multilevel logistic regression model was used to explore influencing factors at the individual and provincial levels. All analyses accounted for complex sample design including clustering, stratification, and sample weights. The mean score of NK among Chinese women aged 18–49 years was 65.1 ± 11.8, and the awareness rate was 20.9% (95% CI: 19.6−22.3%). Among the five dimensions, the awareness rate of food safety was the highest (74.0%, 95% CI: 72.8−75.3%), and the dietary recommendation was the lowest (16.4%, 95% CI: 15.3−17.5%). The two-level logistic regression model analysis revealed that at the individual level, age, education level, occupation, chronic disease status and residence were the main influencing factors of the NK level. At the provincial level, the region and the female illiteracy rate were associated with the NK level. Dependent children ratio, per capita income, and health expenditure were not significantly associated with the NK level. The model accounted for 58.8% of the variance in the NK level. The NK level of Chinese women aged 18–49 years was low. Knowledge of dietary recommendations was weakest. Both individual and provincial-level factors were associated with the NK level. There is an urgent need to strengthen nutrition education actions for enhancing the NK of reproductive-age women effectively to improve their dietary behaviors.

## 1. Introduction

Globally, the burden of disease caused by suboptimal diet is increasing. High sodium, low whole grain, and low fruit intake are major dietary risk factors for death and disability-adjusted life years [1]. Dietary risk factors also result in a variety of public health problems in China, such as deficiencies in essential micronutrients, overweight and obesity, and the prevalence of non-communicable diseases (NCDs) [2,3,4,5,6]. Data from China Nutrition and Health Surveillance 2015–2017 (CNHS) revealed that the intakes of most dietary micronutrients in women aged 18–49 years in China were lower than the recommended dietary vitamin intake (RNI) for Chinese residents [7]. The malnutrition rate was 4.3%, and the overweight and obesity rates were 31.2% and 12.9%, respectively [8]. Women aged 18–49 years are women of reproductive age. Their health status not only concerns them, but also influences pregnancy, pregnancy outcomes, and future generations [9]. The lack of nutrition knowledge (NK) is one of the main reasons for their nutrition-related problems, which can be defined as individual cognitive processes related to food and nutritional information [10]. There was a significant association between the NK of reproductive-age women and their diet [11]. Improving maternal NK would lead to healthier food behavior choices, which can increase maternal health and affect maternal BMI status [12,13,14,15,16]. In addition, women are often the primary caregivers for the family in China [17,18]. Their NK levels are directly related to the diet quality and household food security of the entire family, especially for the next generation [16,19]. Previous studies showed that the caregiver’s NK level was a predictor of dietary practices. Lack of NK among caregivers contributed significantly to poor dietary practices of children under five years of age [11], which was one of the main factors for the high incidence of malnutrition in infants and children [20]. Maternal NK affected food intake in children. Higher NK of the guardians was associated with higher vegetable intake [21]. The lower maternal NK, the lower the child’s dietary adequacy rate [22]. which also affected the occurrence of overweight and obesity [23]. In such a context, the NK of reproductive-age women is essential.

Both the Knowledge-Attitude-Practice model applied at the individual level and the social cognitive theory applied at the interpersonal level consider knowledge as an important influencing factor for behavior change [24]. Based on this premise, improving NK is a key component of changing the dietary behavior and health status of women of childbearing age. Acquiring NK and knowing what and how to eat is the first step in changing eating behavior. This provides strong theoretical evidence to support a focus on nutrition education for reproductive-age women. The form and effectiveness of women’s nutrition education vary by age, education level, occupation, income status, geography (e.g., rural vs. urban), and social context. An effective nutrition education intervention program must be based on a comprehensive understanding of NK levels among women of childbearing age.

Thus, NK has become an important focal point in nutrition policies among Chinese governments and organizations. The Chinese government has pointed out in a series of policies, such as the Outline of Healthy China 2030, National Nutrition Plan (2017−2030), and Health China Action (2019−2030), a need to increase the NK awareness rate among residents with clear target requirements. However, a systematic study of reproductive-age women‘s NK has not been conducted in China yet. Most previous studies have been conducted in localized areas of China, but had insufficient representative data on NK surveys, and evaluation tools were heterogeneous. Our objective was to examine the NK level of Chinese women aged 18–49 years based on nationally representative data from the China Nutrition and Health Knowledge Survey (CNHKS) and to explore possible influencing factors at the individual and provincial levels.

## 2. Materials and Methods

### 2.1. Source of Data

The data in this study came from the CNHKS of Chinese adults in 2021, which was the first nationally representative survey of NK among Chinese adults aged 18−64 years. The sampling plan of the CNHKS was conducted using the national Disease Surveillance Point (DSP) system. Considering the balance of geographical and urban–rural stratification factors and the existing work base, 298 DSPs were selected from 31 provinces (municipalities or autonomous regions) in mainland China. The DSPs were used as the primary sampling unit (PSU) in a multistage sampling framework. Subjects were selected from each PSU using multistage-stratified cluster sampling. Within each PSU, three townships were selected. Two villages or residential areas were selected from each chosen township. Each village selected 60 households randomly, and each surveyed household selected one resident aged 18−64 years. Only subjects living at their residence for a period of ≥6 months during the year were eligible to participate. In addition, they should be able to communicate normally and not have serious intellectual disabilities. Overall, 102,398 participants were enrolled in the survey. After data cleaning, a total of 38,065 females aged 18–49 years were included in this study. All respondents volunteered to participate after fully understanding the content and meaning of the survey.

### 2.2. Data Collection

A standard questionnaire was used for data collection, and the respondents were questioned face-to-face by professionally trained investigators.

The questionnaire, named Chinese Nutrition Health Knowledge Questionnaire for Adults (CNHKQ), was developed by a national project team specifically for use in the CNHKS to assess the NK level of Chinese adults aged 18 years and over. The questionnaire had been validated by experts [25] and evaluated for reliability and validity by a pre-test. The overall CNHKQ showed internal consistency (Cronbach’s α = 0.847). The correlation coefficients between each dimension and the overall questionnaire ranged from 0.542 to 0.839.

The CNHKQ consisted of two parts. The first part included basic demographics about the subjects such as gender, birthdate, education level, occupation, chronic disease status, and the source for obtaining nutritional information. The second part contained 20 items relating to five dimensions of NK: dietary recommendations (four items), nutrient contents (five items), the diet–disease relationship (five items), food choices (five items), and food safety (one item). Scores from each item in each dimension were added to give a dimension score, and the five dimension scores were totaled to give an overall score out of 100. The scores of each dimension were converted into percentages for relative evaluation. We used 75 points as the cut-off value because the evaluation of health literacy in China used 80 as the cut-off value, and the difficulty of NK was higher than health literacy. Therefore, the cut-off value was reduced. When the overall score for each participant was higher than or equal to 75, they were considered to have basic NK. The main index to evaluate the group NK level was the NK awareness rate, which referred to the percentage of all participants with basic NK. The NK awareness rate for each dimension was the percentage of all participants with knowledge in that dimension (with a score higher than 75% of the full score for that dimension).

### 2.3. Explanatory Variables

Variables such as education level (primary school or below, junior high school, senior high school, junior college, bachelor degree or above), occupation (medical and health institutions, food and restaurant industries, education institutions, others), and chronic disease status (no, yes, unclear) were collected using the CNHKQ, and age was calculated by subtracting the survey date from the respondent’s birthdate, which was divided into four groups (18−24, 25−34, 35−44, 45−49 years). In addition, regions (eastern, central and western), residences (urban and rural), health expenditure, per capita income, dependent children ratio, and female illiteracy rates referred to the provincial level rather than individual level, the data of which were from the China Statistical Yearbook 2021 [26] and China Health Care Statistics Yearbook 2022 [27]. Among them, the health expenditure and per capita income were categorized as tertiles (low: ≤*P*_33.3_, medium: *P*_33.3_−*P*_66.7_, high: ≥*P*_66.7_).

### 2.4. Statistical Analyses

The mean and standard deviation (SD) were used to describe continuous variables, and categorical variables were summarized with percentages. The weighted NK awareness rates were presented as ratios and 95% confidence intervals (95% CI). The analysis of the variability of means between groups was performed by independent sample t-test and analysis of variance (ANOVA). Rao-Scott chi-square test was used to compare the differences in NK awareness rates between or among the subgroups.

Since data collection for the CNHKS used a multi-stage complex sampling method with a hierarchical structure (provincial–individual), the distribution of the response variable among individuals was not independent, and if traditional logistic regression was used, it would increase the probability of making a type I error. Therefore, to explore the factors influencing the NK level, we used the two-level logistic regression model, which was one of the statistical methods specifically designed to deal with data with a hierarchical structure. Here, the dependent variable was defined as whether respondents had NK (respondents with a total score of more than 75 were considered to have NK); that is, having NK was defined as “1” or otherwise “0”. First, a two-level logistic regression random intercept empty model was fitted with individuals as level 1 and provinces as level 2. Intraclass correlation coefficient (ICC) was used to determine the clustering of individuals between the province level. The formula for ICC is shown in Equation (1).

(1) $$ICC=\frac{\sigma_{p}^{2}}{\sigma_{p}^{2}+\frac{\pi^{2}}{3}}$$

where $\sigma_{p}^{2}$ indicates the province-level variance, and $\sigma_{i}^{2}$ represents the individual-level variance, which is $\frac{\pi^{2}}{3}$ (approximate to 3.29).

If the statistical test for the random parameter $\sigma_{p}^{2}$ was statistically significant, it indicated that the data were aggregated at the provincial level. Demographic characteristics as one-level variables and socioeconomic variables as two-level variables were incorporated into the model separately. The proportion of regional differences in NK awareness rates explained by different clusters of variables was illustrated by comparing the proportional change in variance (PCV) at the provincial level. The formula for PCV is shown in Equation (2).

(2) $$PCV=\frac{\sigma_{p1-}^{2}\sigma_{p2}^{2}}{\sigma_{p1}^{2}}$$

where $\sigma_{p1}^{2}$ is the variance in the previous model, and $\sigma_{p2}^{2}$ is the variance in the latter model including variables. The odds ratios (OR) with their 95% confidence intervals (95% CI) were calculated in the fixed-effects part of the model, and variables with a *p*-value < 0.05 were considered influencing factors of the NK level.

In this study, to obtain nationally representative results, we calculated sample selection weights for each stage of the sample based on the survey sampling scheme. In addition, post-stratification weights were also assigned by age, gender, and residence when based on data from the Sixth National Population Census of the People’s Republic of China. The final weight was equal to the product of the sampling weight and the post-stratification weights. All statistical analyses were conducted using SAS 9.4 (SAS Institute Inc., Cary, NC, USA). A *p*-value < 0.05 was considered statistically significant.

## 3. Results

### 3.1. Basic Characteristics

A total of 38,065 women of childbearing age were included in this study, among whom 21,468 (56.4%) were from rural areas, 14,284 (37.5%) lived in eastern China, 13,575 (35.7%) were aged 35−44 years, and 23,912 (62.8%) reported having no chronic diseases. More than 60% of participants had a senior high school education or higher (Table 1).

### 3.2. Nutrition Knowledge among Women of Childbearing Age

Table 1 shows the NK among women of childbearing age. The mean ± SD NK score of women of childbearing age was 65.1 ± 11.8. There were significant differences among different groups (*p* < 0.001). Scores increased with the education level (*P*_trend_ < 0.001). Contrarily, although the difference was minor, scores were found to decrease with age (*P*_trend_ < 0.001). Respondents engaged in medical and health institutions scored the highest, and those engaged in the food and restaurant industries scored the lowest. Respondents who lived in urban districts, eastern China, and who did not have chronic diseases scored significantly higher than those in the corresponding groups (*p* < 0.001).

The awareness rate of women of childbearing age was 20.9% (95% CI: 19.6−22.3%). The rate increased with education level (*P*_trend_ < 0.001); participants who had a bachelor’s degree or above had the highest rates (34.4%, 95% CI: 32.1−36.7%). However, the rate decreased with age (*P*_trend_ < 0.001). Additionally, the rates varied with occupation. Those working in medical and health institutions had the highest awareness rate (42.0%, 95% CI: 38.2−45.7%), and those working in the food and restaurant industries had the lowest (14.3%, 95% CI: 11.4−17.2%). In terms of residence, urban women had a higher NK awareness rate than rural women (25.4% vs. 15.1%, respectively). The NK awareness rate of women in the eastern region (25.8%, 95% CI: 23.9−27.6%) was significantly higher than that of women in the central (16.8%, 95% CI: 14.4−19.2%) and western regions (17.6%, 95% CI: 15.3−20.0%). Respondents who did not have chronic diseases had the highest NK awareness rate (23.7%, 95% CI: 22.0−25.3). The differences were statistically significant (*p* < 0.001).

### 3.3. The Nutrition Knowledge Level in Each Dimension

Table 2 provides the results of the NK level in each dimension. There were significant differences in the NK levels among different dimensions (*p* < 0.001). Respondents had the highest scores in the food safety dimension (80.13), followed by the diet–diseases dimension (72.05). The lowest scores were found in the nutrient contents dimension (60.95). In addition, the NK awareness rates of respondents in the five dimensions showed that the awareness rate in the dietary recommendations dimension was the lowest (16.4%, 95% CI: 15.3−17.5%). The awareness rates in the diet–diseases and food safety dimensions exceeded 50%.

### 3.4. Factors Influencing the Nutrition Knowledge Level

Results from the multilevel logistic regression models can be found in Table 3. The empty model result showed that the variance in the random intercept was statistically significant (σ_μ_^2^ = 0.5874, *p* < 0.01), which indicated that NK levels were aggregated at the provincial level. Additionally, about 15% (ICC = 0.15) of the total variation in NK level was due to provincial differences. The ICC result was also suggestive of using a multilevel model rather than a single-level model. When individual variables were included to fit model 1, the difference in NK awareness rates was explained by 30.5% (PCV = 30.5%). Further including provincial variables to fit model 2, the PCV of model 2 showed that 58.5% of the cluster level variation was explained by the variables in the model. Compared with the empty model, two models with explanatory variables significantly reduced the deviance and improve the goodness of fit of the model. Clearly, the AIC results showed that model 2 was the relatively optimal model (34,322 vs. 34,282).

The likelihood of having NK increased with age (45−49 years vs. 18−24 years, [OR = 1.40, 95% CI: 1.25−1.55], *P*_trend_ < 0.001) and education level (bachelor’s degree or above vs. primary school or below, [OR = 4.71, 95% CI: 4.06−5.37], *P*_trend_ < 0.001). Occupation, residence, and chronic disease status had significant effects on the NK level. Compared with general jobs, women engaged in medical and health institutions (OR = 1.96, 95% CI: 1.78−2.14) and education institutions (OR = 1.18, 95% CI: 1.05−1.31) were more likely to have NK. Respondents who reported good health (OR = 1.65, 95% CI: 1.54−1.77) and chronic diseases (OR = 1.22, 95% CI: 1.08−1.37) were more likely to have NK. The odds of having NK were 1.16 times (OR = 1.16, 95% CI: 1.09−1.23) higher for urban women compared with rural women. Additionally, compared to those who lived in the western region, respondents in the central (OR = 1.46, 95% CI: 1.56−2.35) and eastern regions (OR = 1.80, 95% CI: 1.72−2.86) were more likely to have NK. There was a significant negative correlation between the female illiteracy rate and the NK level (*p* < 0.001). However, we did not find a statistical association between the three provincial variables of dependent children ratio, per capita income, and health expenditure and the NK level. There were still significant factors of NK level at the provincial level that were not accounted for.

## 4. Discussion

Using the latest nationally representative data, we found that the mean score of NK among Chinese women aged 18–49 years was 65.1 ± 11.8, only 20.9% of whom had a basic NK level, which is very low. Although national authorities, social organizations and others are constantly taking actions to spread NK, the NK level of women of childbearing age still needs to be improved.

Compared to the other four dimensions, women of childbearing age had the most limited understanding of the dietary recommendation dimension, which is normally designed in the general nutrition knowledge questionnaire [28,29]. It examines adherence to dietary guidelines, including the recommended daily allowance for core foods. Our study found low awareness of the recommended intake levels of core foods such as dairy, soybeans, oil and salt. This is consistent with the findings of another study from CNHKS (n = 98,567) [30]. Data from the CNHS showed that the dairy and soybeans intake of women aged 18–49 years were merely 18.1 g/day and 8.9 g/day, respectively. Both were below the recommended intake levels. However, salt and oil intakes were 8.2 g/day and 39.0 g/day, respectively, which exceeded the recommended intake levels [3]. Several previous studies demonstrated a significant association between NK and healthy eating behaviors; women who had better NK also exhibited better dietary behavior [13,31,32,33], which suggested that the lack of relevant NK among women of childbearing age may be an important reason for their inadequate intake. Increased knowledge about the intake of certain foods may increase their consumption.

The present study also showed that age, education level, residence, and chronic disease status had significant associations with the NK level. Several studies reported similar findings [16,32,33]. Participants engaged in the food and restaurant industries were expected to have higher NK levels. However, contrary to our expected results, they did not show a good NK level, and as relevant people engaged in food production, processing, and sales, their NK levels affected the production of healthy food and consumers’ healthy food choices. Therefore, people in the food and restaurant industries need more training in NK. Moreover, the associations with age, education level, residence, and chronic disease status suggested that young and rural women, especially those with less education, should become the key focus groups for future nutrition education.

Among provincial-level variables, the female illiteracy rate showed a significant negative association with the NK level. Literacy is acquired primarily through formal education and is an important determinant of an individual’s ability to understand and respond to a wide range of health information. A study in the United States showed that limited literacy skills might also severely limit access to NK, choosing and implementing a healthy diet, and making other positive health-related choices [34].

Our study showed that the geographical region was also associated with the NK level. Participants from western areas were less likely to possess NK, which is consistent with our previous report [35]. According to some studies from other countries, mothers who live in socioeconomically disadvantaged areas make food choices that are influenced by low NK levels and limited food resources. They were more likely to choose and purchase what they perceived to be tasty, cheaper foods over healthier foods [11,16,36]. In addition, underdeveloped areas have limited health resources and lack regular nutrition education. Residents access nutritional health information poorly. Although economic conditions constrain underdeveloped areas, studies showed that NK can attenuate socioeconomic differences in food purchasing choices [36]. Improving NK in mothers living in socioeconomically disadvantaged regions improves the home food environment (food availability) and the mother’s and children’s diets [11,19]. Therefore, investing more effort in nutrition education and improving the NK level of women in underdeveloped areas will help to compensate for the impact of socioeconomic differences on the local population’s dietary behavior. Western areas need more robust and targeted efforts in nutritional educational intervention.

NK was linked to household income in previous studies [37,38]. High-income groups are more likely to be concerned about their body image and health, and nutrition and health considerations are more prominent in food choices. Additionally, since we did not collect information about household income, we wanted to use the provincial variable of per capita income as an alternative variable, but the result did not reveal an association with the NK level. This may be because per capita income by province does not accurately reflect household income. The provincial variables of health expenditure and dependent children ratio were also not associated with the NK level in this study. Health expenditure reflects the extent to which the government, society, and individual residents attach importance to health care under certain economic conditions. Although we attempted to demonstrate that the more a region invests in health, the higher the NK level of its residents, the total cost of health involves so many aspects that the impact on NK may be minimal. More detailed indicators may be required to assess a region’s investment in nutrition education actions. Regarding the dependent children ratio, there was evidence that respondents with two or more children at home have significantly higher NK levels than those with no children or only one child [39]. However, the provincial dependent children ratio reflects the number of children and adolescents to be covered per 100 working-age population and is only an average for the region. It is not equivalent to the number of children of the respondents, which could account for the inconsistent findings between the results and expectations.

To the best of our knowledge, this was the first study to assess the NK level of Chinese women of childbearing age and to explore possible influencing factors at the individual and provincial levels using nationally representative survey data. In addition, we used a well-validated NK tool. However, because the data in this study were from a cross-sectional survey, there were significant limitations in exploring causality, as it was not possible to determine causality with cross-sectional data. In addition, due to the lag in relevant publications, the relevant provincial data obtained were not accurate enough, and subsequent studies should have a more comprehensive consideration of the information collected. Nevertheless, our study provides reliable data to assess the current state of NK among women of childbearing age and inform priorities for the precise implementation of nutrition education interventions.

## 5. Conclusions

The NK level of Chinese women of childbearing age is low. Nutrition education for Chinese women of childbearing age should be enhanced—in particular, NK related to dietary guidelines.

Age, education level, occupation, chronic disease status, residence, region, and the female illiteracy rate are the main factors influencing the NK level. Nutrition education should focus on those who are undereducated, unsure of their chronic disease status, work in food and restaurant industries, and live in rural, western areas with high female illiteracy rates.

We propose to implement dynamic monitoring of the NK level of Chinese women using a unified assessment tool to help us continuously improve nutrition education strategies and carry out targeted nutrition actions.

## Figures and Tables

**Table 1:** Univariate analysis of nutrition knowledge (NK) level among participants.

Characteristics	Samples, N (%)	Score (Mean ± SD)	*p*-Value	Awareness Rate (%) (95% CI)	*p*-Value
Overall	38,065	65.1 ± 11.8		20.9 (19.6, 22.3)	
Age (years)			<0.001		<0.001
18−24	8643 (22.7)	65.7 ± 11.2		21.8 (19.5, 24.1)	
25−34	10,768 (28.3)	65.5 ± 11.2		19.9 (17.4, 22.4)	
35−44	13,575 (35.7)	65.2 ± 12.0		21.4 (18.7, 24.0)	
45−49	5079 (13.3)	63.3 ± 12.9		20.6 (17.3, 23.9)	
*P*_trend_		<0.001		<0.001	
Education			<0.001		<0.001
Primary school or below	5226 (13.7)	57.2 ± 13.1		7.5 (6.0, 9.1)	
Junior high school	12,673 (33.3)	62.1 ± 11.4		12.3 (10.8, 13.8)	
Senior high school	7327 (19.2)	65.6 ± 10.9		20.8 (18.7, 22.9)	
Junior college	6948 (18.3)	67.8 ± 10.3		26.4 (24.0, 28.7)	
Bachelor degree or above	5891 (15.5)	70.2 ± 10.5		34.4 (32.1, 36.7)	
*P*_trend_		<0.001		<0.001	
Occupation			<0.001		<0.001
Medical and health institutions	3212 (8.4)	71.2 ± 10.5		42.0 (38.2, 45.7)	
Food and restaurant industries	2579 (6.8)	62.8 ± 11.6		14.3 (11.4, 17.2)	
Education institutions	2336 (6.1)	67.1 ± 10.5		24.4 (21.0,27.8)	
Others	29,938 (78.6)	64.3 ± 11.8		18.3 (17.1, 19.5)	
Residence			<0.001		<0.001
Urban	16,597 (43.6)	66.9 ± 11.2		25.4 (22.7, 28.1)	
Rural	21,468 (56.4)	62.7 ± 12.0		15.1(12.7, 17.6)	
Region			<0.001		<0.001
Eastern	14,284 (37.5)	67.2 ± 11.3		25.8 (23.9, 27.6)	
Central	10,659 (28.0)	64.1 ± 11.2		16.8 (14.4, 19.2)	
Western	13,122 (34.5)	62.9 ± 12.5		17.6 (15.3, 20.0)	
Chronic diseases			<0.001		<0.001
No	23,912 (62.8)	66.4 ± 11.2		23.7 (22.0, 25.3)	
Yes	3522 (9.3)	63.6 ± 12.7		19.5 (16.4, 22.6)	
Unclear	10,631 (27.9)	62.7 ± 12.1		15.2 (13.5, 16.9)	

**Table 2:** Participants’ nutrition knowledge levels in each dimension.

Dimension	Full Scores	Scores (Mean ± SD)	Standard Score	Awareness Rate (%) (95% CI)
Dietary recommendations	30	18.39 ± 4.09	61.30	16.4 (15.3, 17.5)
Nutrient contents	21	12.80 ± 3.23	60.95	19.5 (18.3, 20.6)
Diet–diseases	22	15.85 ± 3.97	72.05	51.3 (49.7, 52.8)
Food choices	19	11.67 ± 3.13	61.42	21.4 (20.4, 22.4)
Food safety	8	6.41 ± 1.96	80.13	74.0 (72.8, 75.3)
*p*-value		<0.001	<0.001	<0.001

**Table 3:** Factors associated with NK level: fixed and random effects parameters estimated from multilevel logistic regression [OR (95% CI)].

	Empty Model	Model 1 Model with Individual Variables	Model 2 Model with All Variables
Fixed effects			
Age (years) (ref: 18–24)			
25−34		1.12 (1.03, 1.20) *	1.11 (1.02,1.19) *
35−44		1.32 (1.21, 1.42) *	1.29 (1.19, 1.39) *
45−49		1.45 (1.30, 1.60) *	1.40 (1.25, 1.55) *
Education (ref: Primary School or Below)			
Junior high school		1.74 (1.52, 1.95) *	1.69 (1.49, 1.90) *
Senior high school		2.71 (2.36, 3.06) *	2.56 (2.23, 2.90) *
Junior college		3.63 (3.15, 4.11) *	3.36 (2.91, 3.82) *
Bachelor degree or above		5.17 (4.47, 5.87) *	4.71 (4.06, 5.37) *
Occupation (ref: Others)			
Food and restaurant industries		1.01 (0.89, 1.13)	1.01 (0.89, 1.13)
Education institutions		1.18 (1.05, 1.31) *	1.18 (1.05, 1.31) *
Medical and health institutions		1.96 (1.78, 2.13) *	1.96 (1.78, 2.14) *
Chronic diseases (ref: Unclear)			
No		1.65 (1.54, 1.77) *	1.65 (1.54, 1.77) *
Yes		1.23 (1.08, 1.37) *	1.22 (1.08, 1.37) *
Residence (ref: Rural)			
Urban		1.16 (1.09, 1.24) *	1.16 (1.09, 1.23) *
Region (ref: Western)			
Central			1.46 (1.56, 2.35) *
Eastern			1.80 (1.72, 2.86) *
Health expenditure (ref: Low)			
Medium			1.29 (0.81, 1.76)
High			1.62 (0.80, 2.43)
Per capita income (ref: Low)			
Medium			0.87 (0.48, 1.25)
High			1.39 (0.41, 2.36)
Female illiteracy rate (%) ^1^			0.92 (0.88, 0.95) *
Dependent children ratio (%) ^2^			1.00 (0.97, 1.03)
Random effects			
σ_p_^2^ (standard error)	0.59 (0.17) *	0.41 (0.12) *	0.17 (0.05) *
PCV (%)	reference	30.5%	58.5%
ICC	0.15	0.11	0.05
AIC	36,274	34,299	34,282

^1^ Percentage of illiterate women to total aged 15 and over by province; ^2^ Ratio of people aged 0−14 to people aged 15−64 by province; * *p*-value < 0.001. Model 2 further incorporates provincial variables, including region, the health expenditure, per capita income, dependent children ratio, and female illiteracy rate. Abbreviation: AIC, Akaike information criterion.

## Data Availability

The data presented in this study are not public.
